# Correlates of Caregiver Burden Among Informal Caregivers of Patients With Hematological Malignancies: The Roles of Coping, Resilience, and Perceived Social Support

**DOI:** 10.7759/cureus.116477

**Published:** 2026-09-18

**Authors:** Nasrin Akhter, Amin Lutful Kabir, Adnan Hasan Masud, Kazi Maksoda Akter, Kazi Mohammad Kamrul Islam, Bilkis Ferdous, Shamsun Nahar, Tahmidul Islam, Nusaiba Jasmine, Nazirum Mubin

**Affiliations:** 1 Department of Hematology, Bangladesh Medical University, Dhaka, BGD; 2 Department of Palliative Medicine, Bangladesh Medical University, Dhaka, BGD; 3 Department of Obstetrics and Gynecology, Bangladesh Medical University, Dhaka, BGD; 4 Department of Clinical Oncology, Bangladesh Medical University, Dhaka, BGD; 5 Department of Biochemistry, Bangladesh Medical University, Dhaka, BGD; 6 Department of Radiotherapy, Dhaka Medical College Hospital, Dhaka, BGD; 7 Department of Non-communicable Diseases, Centre for Medical Research & Development (CMRD), Dhaka, BGD

**Keywords:** bangladesh, caregiver burden, coping, hematological malignancies, perceived social support, resilience

## Abstract

Background

Informal caregivers of patients with hematological malignancies often face prolonged physical, psychological, and practical demands; however, evidence regarding modifiable correlates of caregiver burden remains limited in Bangladesh. This study aimed to determine the level of caregiver burden and examine the relationships of coping strategies, resilience, perceived social support, and caregiving-related factors with burden among informal caregivers.

Methods

This single-center, hospital-based cross-sectional study was conducted in the Department of Hematology, Bangladesh Medical University, Dhaka, from February to May 2026. Sixty-five adult primary informal caregivers who had provided care for at least three months were recruited consecutively. Caregiver burden, coping ability, resilience, and perceived social support were assessed via the Bangla Zarit Burden Interview (ZBI), Brief COPE, Connor-Davidson Resilience Scale (CD-RISC-25), and Multidimensional Scale of Perceived Social Support (MSPSS), respectively. The data were analyzed via one-way analysis of variance, Pearson’s correlation, and multiple linear regression in IBM SPSS Statistics for Windows, Version 22 (Released 2013; IBM Corp., Armonk, New York, United States). Regression assumptions, including linearity, normality and homoscedasticity of residuals, multicollinearity and influential observations, were examined before the adjusted estimates were interpreted.

Results

Of the 65 caregivers, 28 (43.1%) had moderate-to-severe burdens, and 10 (15.4%) had severe burdens; overall, 38 (58.5%) experienced moderate-to-severe or severe burdens. Problem-focused coping decreased significantly across increasing burden categories (p = 0.020). Caregiver burden was inversely correlated with problem-focused coping (r = −0.430, p<0.001) and resilience (r = −0.412, p<0.001). In this model, longer total caregiving duration was independently associated with greater burden (B = 1.367, 95% CI 0.101 to 2.633, β = 0.217, p = 0.035), whereas problem-focused coping was independently associated with lower burden (B = −2.330, 95% CI −3.343 to −1.317, β = −0.789, p<0.001). Emotion-focused coping (B = 1.121, 95% CI 0.290 to 1.952, β = 0.446, p = 0.009) and perceived social support (B = 0.251, 95% CI 0.067 to 0.435, β = 0.328, p = 0.008) showed positive adjusted associations with burden. Daily caregiving hours, dysfunctional coping, and resilience were not independently associated with burden.

Conclusion

Caregiver burden was substantial in this population. A longer duration of caregiving and coping patterns were important correlates, whereas the unexpected positive adjusted association with perceived social support requires cautious interpretation and should not be read as evidence that social support increases burden. Because the design was cross-sectional, all findings describe associations rather than causal determinants. Routine caregiver screening and culturally appropriate support focused on practical coping and prolonged caregiving demands may be beneficial.

## Introduction

Hematological cancers encompass several biologically distinct disorders, such as leukemia, lymphoma, multiple myeloma, and myelodysplastic syndromes, that result from abnormalities in blood cell production. Global Cancer Observatory (GLOBOCAN) 2022 estimated 1,311,104 incident cases and 700,205 deaths globally, with non-Hodgkin lymphoma accounting for the largest share [1]. Although diagnostic and therapeutic progress has extended survival in several subtypes, many patients still follow a prolonged course marked by recurrence and repeated outpatient treatment rather than a single episode of care [2]. Clinical trajectories and prognoses therefore differ substantially among subtypes.

During the illness trajectory, informal family caregivers coordinate hospital attendance, monitor symptoms and medicines, support infection prevention and daily activities, and often manage financial responsibilities. Those caring for patients receiving hematopoietic stem-cell transplantation (HSCT) face considerable demands, psychological distress, and poorer quality of life, partly because disease progression is uncertain and hospital admissions may recur [3].

Caregiver burden denotes the multidimensional physical, emotional, social, occupational, and financial strain of unpaid caregiving, a concept first articulated among caregivers of impaired elderly individuals [4]. Among caregivers of hemato-oncological patients, a substantial proportion report high psychological burdens alongside significant depressive symptoms, which are related but distinct constructs [2]. Similar patterns emerge among caregivers of patients with advanced hematological malignancies, over half of whom report a high burden with impaired quality of life [5]. Caregiving here has also been linked with fatigue, sleep disturbance, and social isolation.

Coping, within the classical stress-appraisal framework, denotes cognitive and behavioral efforts to manage appraised demands [6]. Problem-focused coping is associated with decreased burden and better adjustment, whereas emotion-focused coping has a variable relationship with outcomes and should not be uniformly labeled maladaptive [7]. Among HSCT caregivers, approach-oriented coping is linked with lower distress, whereas avoidant coping is linked with greater anxiety, depression, and poorer quality of life [3].

Resilience refers to the ability to maintain psychological functioning despite adversity and may be particularly important when caregiving is prolonged and uncertain. In families caring for patients with advanced cancer, psychological distress appears to explain part of the link between burden and quality of life, whereas family resilience changes the strength of that pathway [8]. Resilience may therefore represent a modifiable resource, although its contribution among caregivers of people with hematological malignancies remains insufficiently understood.

Perceived social support reflects a caregiver's belief that assistance is available from family, friends, or healthcare professionals. Among caregivers of patients awaiting HSCT, lower perceived support is associated with greater burden [9]. This relationship is not straightforward: perceived support does not always match the support received, and highly burdened caregivers may seek more support while finding it insufficient.

Informal caregiving can be particularly taxing in low- and middle-income settings because formal respite options are scarce, family members provide the most care, expenses are often paid out of pocket, and health worker shortages add pressure. Evidence from a tertiary palliative-care service in Bangladesh documented considerable burdens among relatives caring for people with advanced cancer; the caregiver's relationship with the patient and knowledge of the prognosis were among the associated factors. The scarcity of organized caregiver support programs may intensify these pressures further [10].

Although caregiver burden has been widely investigated in general oncology, evidence focused on caregivers of patients with hematological malignancies is sparse, and coping, resilience, and social support have usually been examined separately [5]. This evidence gap is especially important in Bangladesh, where relatives deliver extensive unpaid care without routine screening, education, or psychosocial assistance [10]. Clarifying modifiable contributors to burdens could guide screening, coping-skill training, resilience-oriented support, and family-centered hematology services suited to the local culture. Accordingly, this study assessed the extent of caregiver burden and examined coping patterns, resilience, perceived social support, and caregiving characteristics as potentially modifiable correlates, rather than causal determinants, among informal caregivers of Bangladeshi patients with hematological malignancies.

## Materials and methods

Design and study site

An observational single-center cross-sectional survey was undertaken at the Department of Hematology, Bangladesh Medical University, Dhaka. This tertiary academic referral center provides specialized diagnostic and treatment services for hematological disorders and cancers. Recruitment and data collection spanned four months, from February through May 2026.

Study participants

The study population comprised adult primary informal caregivers of patients diagnosed with hematological malignancies who were receiving active treatment at the study center. A primary informal caregiver was considered an unpaid family member or close associate who assumed the principal responsibility for providing day-to-day assistance, accompanying the patient during treatment, and supporting the patient’s physical, practical, or emotional needs. Only one primary caregiver was enrolled for each patient.

Eligibility criteria

Eligibility required informal caregivers to be at least 18 years old, identify as the patient's main caregiver, provide care for three months or longer, understand and complete the questionnaires, and provide written informed consent. Individuals with severe cognitive impairment, communication problems, or other conditions that made reliable questionnaire completion impossible were excluded.

Sampling and sample size calculation

The sample size was estimated with a single-proportion formula using the prevalence of moderate-to-severe caregiver burden reported previously. Mishra et al. reported that 21.38% of the relatives of patients receiving chemotherapy had moderate-to-severe burdens according to the Zarit Burden Interview (ZBI) [11]. With a 95% confidence level ($Z_{\alpha/2} = 1.96$), an expected proportion of p = 0.2138, and an absolute precision of d = 0.10, the sample size was calculated using the following formula:

\[n = \frac{Z_{\alpha/2}^{2}\,p(1-p)}{d^{2}}\]

Accordingly, \[n = \frac{(1.96)^{2}\times 0.2138\times 0.7862}{(0.10)^{2}} = 64.57\]

The calculation yielded a minimum of 65 caregivers after rounding. Consecutive nonprobability recruitment was used because a complete list of eligible informal caregivers was unavailable. Potential participants attending the Department of Hematology were approached in sequence throughout the study period until 65 eligible caregivers were enrolled. The single-proportion formula was used because the primary objective of the study was to estimate the prevalence of moderate-to-severe caregiver burden in this population, for which a published proportion was available; the multiple linear regression model was a secondary and exploratory analysis and was not the basis of the sample-size calculation. With 65 participants and seven independent variables, the ratio of approximately nine observations per variable falls below the 10 to 15 observations per variable that are commonly recommended for stable multivariable linear regression. A post hoc calculation indicated that this sample provided approximately 80% power at a two-sided α of 0.05 to detect an overall model effect of f² ≈ 0.25 (equivalent to R² ≈ 0.20) with seven independent variables, but limited power to detect small individual associations. The study was therefore adequately powered for the prevalence estimate and for a moderate overall model effect, but not for precise estimation of individual adjusted coefficients.

Data collection procedure

Information was obtained at one face-to-face study visit via a predesigned structured questionnaire together with four standardized psychometric measures. Once eligibility was verified, researchers explained the study purpose and procedures, privacy protections, and the optional nature of participation. The participants then provided written informed consent before assessment.

The structured questionnaire captured caregiver age, gender, marital status, education, occupation, household income, and comorbidities. It also recorded the caregiver's relationship with the patient, typical hours of care per day, and total time spent in the caregiving role. Burden, coping, resilience, and perceived support were measured with the ZBI, Brief COPE, Connor-Davidson Resilience Scale (CD-RISC-25), and Multidimensional Scale of Perceived Social Support (MSPSS), respectively. Before data entry, each completed form was checked for missing or inconsistent responses.

Measurement of caregiver burden

Caregiver burden was measured with the validated 22-item Bangla ZBI, an instrument originally designed to quantify perceived burden among those caring for older adults with cognitive impairment or dementia [4,12]. Responses are scored from 0 ("never") to 4 ("nearly always") on a five-point Likert scale, giving a possible total of 0-88; larger totals denote heavier burdens. Scores of 0-20 indicate little or no burden, 21-40 indicate mild-to-moderate burden, 41-60 indicate moderate-to-severe burden, and 61-88 indicate severe burden. These severity bands are the conventional interpretive categories of the original 22-item ZBI and were applied without modification. The Bangla adaptation [12] established the linguistic and clinimetric equivalence, internal consistency, and test-retest reliability of the 22-item instrument but did not derive Bangla-specific cut-off scores; because the item set, the 0 to 4 response format, and the 0-88 total range are identical in the two versions, the original bands were retained, with the 20 to 21 boundary presented as mutually exclusive so that every score falls into exactly one category. Earlier testing of the Bangla version revealed good internal consistency and test-retest reliability [12]. In this sample, internal consistency was acceptable (Cronbach's α = 0.795).

Measurement of coping ability

Coping was evaluated with the validated 28-item Bangla Brief COPE, which contains 14 subscales of two items each [13]. The four response options range from 1 ("I have not been doing this at all") to 4 ("I have been doing this a lot"); higher values indicate more frequent use of a given strategy. For analysis, the original subscales were combined into problem-focused, emotion-focused, and dysfunctional domains according to the three-component model proposed by Cooper et al. [14]. Following that model, the problem-focused domain comprised active coping, planning, and use of instrumental support (three subscales, six items, possible range 6-24); the emotion-focused domain comprised acceptance, use of emotional support, humor, positive reframing, and religion (five subscales, ten items, possible range 10-40); and the dysfunctional domain comprised behavioral disengagement, denial, self-distraction, self-blame, substance use, and venting (six subscales, twelve items, possible range 12-48). Each domain score was calculated as the unweighted sum of the raw item scores of its constituent subscales, and the total coping score was the sum of all 28 items (range 28-112); no items were reverse-scored, and no mean-based or standardized scoring was applied. The Bangla instrument has previously shown satisfactory factorial validity and internal consistency [15]. The Cronbach's α for the scale in the current sample was 0.738, indicating acceptable internal consistency.

Measurement of resilience

The 25-item CD-RISC-25, developed by Connor and Davidson [16], was used to measure resilience. Items are scored from 0 ("not true at all") to 4 ("true nearly all of the time"), producing a total score between 0 and 100; higher totals reflect stronger resilience. The participants completed the scale in Bangla. Its internal consistency in this study was good (Cronbach's α = 0.878).

Measurement of perceived social support

Perceived support was measured with the validated 12-item Bangla MSPSS, originally developed by Zimet et al. [17,18]. Its three four-item subscales assess support from family, friends, and a significant other. Each statement is rated from 1 ("very strongly disagree") to 7 ("very strongly agree"), so total scores range from 12 to 84; higher values indicate stronger perceived support. Validation of the Bangla version confirmed the original three-factor structure and showed satisfactory reliability and construct validity [18]. The internal consistency of the present sample was excellent (Cronbach's α = 0.932). For descriptive reporting, family, friend, significant other, and overall scores were divided into sample-specific quartiles, with successively higher quartiles denoting greater perceived support.

Data analysis

Data were analyzed with IBM SPSS Statistics for Windows, Version 20 (Released 2013; IBM Corp., Armonk, New York, United States). Normally distributed continuous variables are presented as means ± standard deviations, skewed variables as medians with interquartile and observed ranges, and categorical variables as frequencies and percentages. Coping domain and total coping scores were compared across burden categories using one-way analysis of variance. Perceived social support was summarized in sample-specific quartiles, while the continuous MSPSS total was used in correlation and regression. Pearson correlation related the total ZBI score to daily caregiving hours, total caregiving duration, each coping domain, resilience, and perceived social support. Multiple linear regression followed, with the total ZBI score as the dependent variable and these seven variables entered simultaneously as independent variables. Model assumptions were examined before interpretation. Linearity and homoscedasticity were assessed from residual and partial regression plots, normality of residuals from histograms, normal probability plots, and the Shapiro-Wilk test, independence from the Durbin-Watson statistic, multicollinearity from tolerance and variance inflation factors (tolerance below 0.10 or VIF above 5 indicating a problem), and influential observations from Cook's distance (values above 1). Model fit is reported as R², adjusted R², and the F statistic with its degrees of freedom, and 95% confidence intervals are given for all unstandardized coefficients. Because a cross-sectional design cannot establish temporality, the findings are described as independent associations rather than predictions. All tests were two-sided, with p < 0.05 indicating statistical significance.

Ethical approval and consent

Ethical approval was obtained from the Institutional Review Board of Bangladesh Medical University (approval no: BMU/2026/884). The study was conducted in accordance with the ethical principles of the Declaration of Helsinki. The participants received information about the study purpose, voluntary participation, confidentiality, and freedom to withdraw from the study at any time without penalty. Written informed consent was obtained from all participants before any data were collected.

## Results

The analyses included 65 informal caregivers of patients with hematological malignancies. The largest age group was ≤30 years (27, 41.5%), followed by 31-40 years (23, 35.4%), >50 years (10, 15.4%), and 41-50 years (5, 7.7%). There were 39 women (60.0%) and 26 men (40.0%). Forty-eight (73.8%) were married, and 34 (52.3%) reported a monthly income of ≤20,000 BDT. Eleven caregivers (16.9%) had comorbidities. Twenty-three (35.4%) had completed HSC or higher education, and housewives composed the largest occupational group (29, 44.6%). The average duration of care was 11.06 ± 4.82 hours daily; the median total duration was 1.0 years (IQR 1.0; range, 0.25-16 years) (Table 1).

**Table 1:** Sociodemographic profile and caregiving responsibilities of the participants (N = 65) The values are reported as n (%), means ± standard deviations, medians (interquartile ranges), or observed ranges, as applicable. BDT: Bangladeshi Taka; SSC: secondary school certificate; HSC: higher secondary certificate; SD: standard deviation; IQR: interquartile range

Characteristics	Category	Frequency (n)	Percentage (%)
Age (years)	≤30	27	41.5
	31–40	23	35.4
	41–50	5	7.7
	>50	10	15.4
Gender	Male	26	40.0
	Female	39	60.0
Marital status	Married	48	73.8
	Unmarried	17	26.2
Monthly income (BDT)	≤20,000	34	52.3
	21,000–40,000	23	35.4
	>40,000	8	12.3
Comorbidity	Present	11	16.9
	Absent	54	83.1
Educational status	No formal education	7	10.8
	Primary	15	23.1
	SSC	20	30.8
	HSC and above	23	35.4
Occupation	Housewife	29	44.6
	Student	14	21.5
	Service holder	13	20.0
	Business	4	6.2
	Day labor	3	4.6
	Unemployed	2	3.1
Duration of caregiving (hours/day)	Mean ± SD	11.06 ± 4.82	
Total duration of caregiving (years) Median (IQR)	1.0 (1.0)	Min– Max (0.25 – 16)	

One caregiver (1.5%) reported little or no burden, and 26 (40.0%) had mild-to-moderate burdens. The burden was moderate-to-severe in 28 (43.1%) caregivers and severe in 10 (15.4%) caregivers. When the upper two categories were combined, 38 caregivers (58.5%) had at least moderate-to-severe burdens (Figure 1).

**Figure 1:**
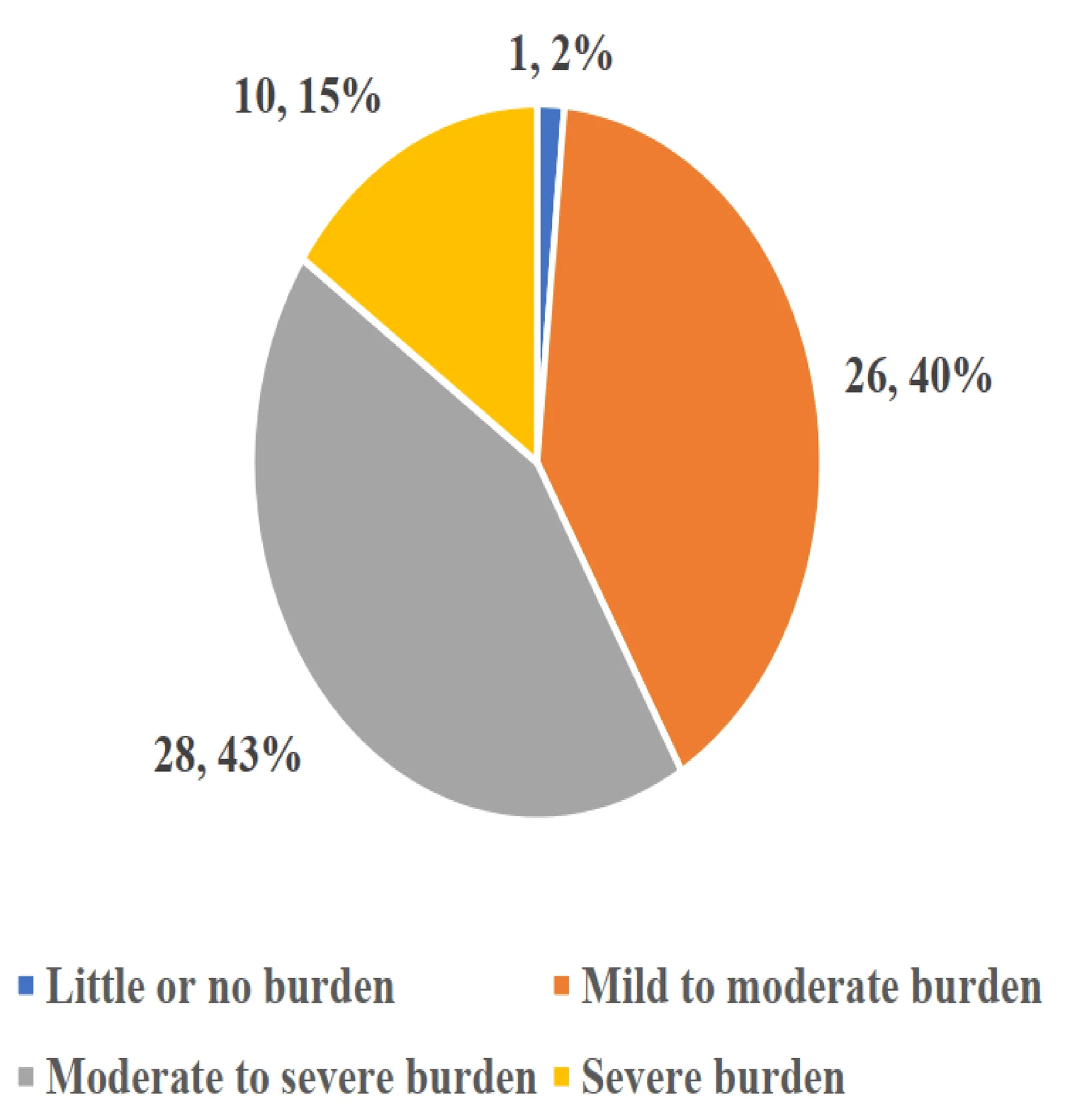
Caregiver-burden categories among participants (N = 65)

Problem-focused coping declined as burden severity increased: 24.00 ± 0.00 for little or no burden, 21.69 ± 3.16 for mild-to-moderate burden, 19.68 ± 3.94 for moderate-to-severe burden, and 18.00 ± 2.79 for severe burden. The between-group difference was significant (p = 0.020). The emotion-focused, dysfunctional, and total coping scores did not vary significantly by burden category (all p > 0.05) (Table 2).

**Table 2:** Coping scores according to caregiver-burden category (N = 65) The values are the means ± standard deviations. One-way analysis of variance was used to compare problem-focused, emotion-focused, dysfunctional, and total coping scores among burden categories; p < 0.05 indicated statistical significance. F statistics are reported with (3, 61) degrees of freedom.

Coping type	Little or no burden	Mild to moderate	Moderate to severe	Severe burden	F statistic	p-value
Problem-focused	24.00±0.00	21.69±3.16	19.68±3.94	18.00±2.79	3.51	0.020
Emotion-focused	38.00±0.00	37.15±5.36	38.25±3.91	37.70±2.63	0.28	0.841
Dysfunctional	26.00±0.00	22.38±4.69	23.89±5.99	23.40±3.86	0.48	0.699
Coping score (total)	88.00±0.00	81.23±10.88	81.82±9.71	79.10±6.10	0.35	0.788

Caregivers had a mean resilience score of 59.80 ± 13.96; the observed values ranged from 20.00 to 81.00. Table 3 summarizes groups of caregivers by sample-derived quartiles of perceived social support. For the total score, 16 (24.6%) fell into the first quartile, 18 (27.7%) into the second quartile, 17 (26.2%) into the third quartile, and 14 (21.5%) into the fourth quartile.

**Table 3:** Sample-based quartiles of perceived social support (N = 65) The family, friend, significant other, and total MSPSS scores were divided into quartiles. Values are n (%). MSPSS: Multidimensional Scale of Perceived Social Support

Parameters	Quartile	Frequency (n)	Percentage (%)
Support from family	1^st^	18	27.7
	2^nd^	15	23.1
	3^rd^	16	24.6
	4^th^	16	24.6
Support from friends	1^st^	18	27.7
	2^nd^	18	27.7
	3^rd^	17	26.2
	4^th^	12	18.5
Support from significant other	1^st^	16	24.6
	2^nd^	18	27.7
	3^rd^	16	24.6
	4^th^	15	23.1
Total perceived social support	1^st^	16	24.6
	2^nd^	18	27.7
	3^rd^	17	26.2
	4^th^	14	21.5

Caregiver burden was inversely correlated with problem-focused coping (r = −0.430, p < 0.001) and resilience (r = −0.412, p < 0.001). Its correlation with total duration of caregiving was weakly positive but not statistically significant (r = 0.222, p = 0.075). Daily caregiving hours, emotion-focused coping, dysfunctional coping, and perceived social support were not significantly correlated with burden (all p > 0.05) (Table 4).

**Table 4:** Bivariate relationships between caregiver burden and caregiving or psychosocial measures (N = 65) Pearson's r was calculated between the total ZBI score and each selected independent variable. Positive values indicate direct relationships, and negative values indicate inverse relationships; p < 0.05 was considered statistically significant. MSPSS: Multidimensional Scale of Perceived Social Support; ZBI: Zarit Burden Interview

Variable	Pearson's *r*	p-value
Duration of caregiving (hours/day)	0.035	0.780
Total duration of caregiving (years)	0.222	0.075
Problem-focused coping	-0.430	<0.001
Emotion-focused coping	0.020	0.873
Dysfunctional coping	0.073	0.563
Resilience	-0.412	<0.001
Perceived social support (MSPSS)	0.036	0.779

The adjusted linear regression model was statistically significant and explained 48.6% of the variance in caregiver burden (R² = 0.486, adjusted R² = 0.423; F(7, 57) = 7.70, p < 0.001). In this model, a longer total duration of caregiving was independently associated with greater burden (B = 1.367, 95% CI 0.101 to 2.633, β = 0.217, p = 0.035), whereas stronger problem-focused coping was independently associated with lower burden (B = −2.330, 95% CI −3.343 to −1.317, β = −0.789, p < 0.001). Emotion-focused coping (B = 1.121, 95% CI 0.290 to 1.952, β = 0.446, p = 0.009) and perceived social support (B = 0.251, 95% CI 0.067 to 0.435, β = 0.328, p = 0.008) were also independently associated with greater burden. Daily hours of care, dysfunctional coping, and resilience were not independently associated with burden (all p > 0.05) (Table 5). Model diagnostics supported the use of the model, although the estimates remain imprecise. Multicollinearity was not evident, with tolerance values ranging from 0.31 to 0.89 and variance inflation factors from 1.12 to 3.26, all below the threshold of 5. Inspection of the residual scatterplots and normal probability plots showed no material departure from linearity, homoscedasticity, or normality; the Shapiro-Wilk test of the residuals was non-significant; the Durbin-Watson statistic was within the acceptable range of 1.5 to 2.5; and no observation exceeded a Cook's distance of 1.

**Table 5:** Adjusted linear regression model for caregiver burden (N = 65) In this model, the total ZBI score was the outcome variable. The estimates are shown as unstandardized coefficients (B), standard errors (SEs), 95% confidence intervals (CIs) for B, standardized coefficients (β), t, and p. Positive coefficients denote increasing burdens as the independent variable increases; negative coefficients denote an inverse relationship. Statistical significance was set at p < 0.05. Because the study was cross-sectional, the coefficients represent independent associations and not causal effects. MSPSS: Multidimensional Scale of Perceived Social Support; ZBI: Zarit Burden Interview

Variables	B	SE	β	t-value	p-value	95% CI for B
Duration of caregiving (hours/day)	0.293	0.286	0.104	1.024	0.310	−0.280 to 0.866
Total duration of caregiving (years)	1.367	0.632	0.217	2.162	0.035	0.101 to 2.633
Problem-focused coping	-2.330	0.506	-0.789	-4.605	<0.001	−3.343 to −1.317
Emotion-focused coping	1.121	0.415	0.446	2.699	0.009	0.290 to 1.952
Dysfunctional coping	-0.177	0.255	-0.072	-0.692	0.492	−0.688 to 0.334
Resilience	-0.188	0.121	-0.193	-1.550	0.127	−0.430 to 0.054
Perceived social support (MSPSS score)	0.251	0.092	0.328	2.740	0.008	0.067 to 0.435

## Discussion

In this study, more than half experienced moderate-to-severe or severe burdens. At the bivariate level, both problem-focused coping and resilience were inversely related to burden. After adjustment, burdens were greater with longer durations of caregiving, greater emotion-focused coping, and greater perceived social support, but lower with stronger problem-focused coping. Resilience, daily hours of care, and dysfunctional coping did not retain independent associations.

More than half of the participants were classified as having moderate-to-severe or severe burdens on the ZBI. This pattern resembles findings in caregivers supporting HSCT, among whom high burden, distress, and impaired quality of life have been documented during treatment [3]. Substantial burdens have also been described in caregivers of adults with β-thalassemia major in China [19] and children with acute lymphoblastic leukemia in India [20]; however, comparisons should account for differences in disease, treatment stage, and measurement tools. Long and uncertain treatment pathways, together with infection-related isolation, may intensify caregiving demands. In Bangladesh, reliance on relatives, travel for treatment, out-of-pocket expenses, and limited formal services may further increase strain, echoing evidence from local palliative-care settings [10].

Across burden-group comparisons, correlations, and adjusted analyses, stronger problem-focused coping consistently accompanied a lower burden. This finding is consistent with evidence from HSCT caregivers that approach-oriented coping is linked to less distress and better quality of life [3] and with a Brazilian study that reported an inverse association between problem-focused coping and burden in cancer caregivers [21]. Practical problem-solving and coordination of treatment may improve a caregiver's sense of control over manageable tasks. These strategies may be less useful for uncontrollable events such as relapse, so their apparent benefit should not be interpreted as universal.

The positive adjusted association between emotion-focused coping and burden, despite the absence of an unadjusted correlation, is offered here as one possible hypothesis rather than as an explanation established by the present data. One possibility is a reverse direction, in which caregivers who are already highly burdened turn more often to emotion-focused strategies, rather than these strategies increasing burden. Because the design is cross-sectional, temporality cannot be determined, and neither direction can be excluded. A suppression effect arising from the shared variance between problem-focused and emotion-focused coping within the adjusted model is a further possible explanation. Emotion-focused coping is heterogeneous: a study of spouse caregivers of older cancer patients revealed that acceptance was negatively correlated with distress, whereas avoidant strategies were positively correlated with distress [22], indicating that adaptiveness depends on the specific strategy used. Dysfunctional coping was not significantly associated with burden here, which contrasts with mixed findings among cancer caregivers in Vietnam [23].

Resilience showed a moderate inverse correlation with burden, which is consistent with a study of advanced cancer caregivers that linked resiliency to lower burden [24] and with systematic review evidence linking greater resilience to lower distress among adult cancer caregivers [25]. However, resilience did not remain significant after adjustment, suggesting that its bivariate association may overlap with problem-focused coping or other psychosocial characteristics already in the model, not that resilience is clinically unimportant.

Bivariate analysis did not link perceived social support to caregiver burden; after covariate adjustment, however, the association became positive. This direction was unexpected because prior research generally suggests that more social support accompanies less caregiver burden [26,27]. This finding should not be interpreted as evidence that social support increases caregiver burden. Possible explanations include greater mobilization of support for caregivers who are already heavily burdened, support that is emotional rather than practically useful, and statistical overlap among coping, resilience, and support, which is consistent with work on social connectedness in burden-prediction models [28]. Reverse causation, residual confounding, and statistical instability in a small sample are equally plausible accounts, and the direction of this association therefore remains uncertain. The cross-sectional design and small sample mean that these results should be interpreted carefully.

A longer duration of caregiving was independently associated with greater burden, which is consistent with the stress‒process framework, in which cumulative demands may contribute to role restrictions and financial strain over time [29], and with an Indonesian study linking longer duration of care to greater burden and poorer health status among cancer patient caregivers [30]. Daily caregiving hours showed no significant association. A non-significant association does not, however, demonstrate that cumulative duration is more important than daily caregiving intensity. Limited statistical power, measurement error in self-reported daily caregiving hours, and residual confounding may equally explain this result, so the comparison between the two exposures should be regarded as inconclusive. Unmeasured disease severity, treatment phase, and patient dependency may confound this relationship and warrant a longitudinal study.

This investigation is among the few Bangladeshi studies to assess caregiver burden in relatives of patients with hematological malignancies while examining coping, resilience, and perceived support together. Standardized psychometric tools showed acceptable to excellent internal consistency, and multivariable linear regression added analytical strength. Several limitations remain. Because the data came from one center and only 65 participants, the regression estimates may be unstable, and the findings may not be generalizable broadly. In particular, entering seven independent variables in a model with 65 observations gives approximately nine observations per variable, which is below the 10 to 15 observations per variable usually recommended and raises the possibility of overfitting and unstable coefficients. The wide 95% confidence intervals around several estimates reflect this imprecision, and the adjusted findings should therefore be treated as exploratory and hypothesis-generating rather than confirmatory. The cross-sectional design cannot establish the timing or direction of observed relationships. Consecutive nonprobability recruitment may have created selection bias. All psychosocial measures were self-reported during one encounter, resulting in potential recall, social desirability, and common-method bias. In addition, the model did not include malignancy subtype, disease stage, treatment phase, symptom severity, or patient dependency; these unmeasured clinical characteristics could plausibly influence both caregiver burden and the psychosocial variables examined, and they are therefore an important source of residual confounding that may partly account for the observed associations, including the unexpected positive association with perceived social support.

## Conclusions

A substantial caregiver burden was evident among the relatives of patients with hematological malignancies. Greater burden was independently associated with longer duration of caregiving, weaker problem-focused coping, stronger emotion-focused coping, and greater perceived social support; resilience was inversely related only in bivariate analysis. Because the study was cross-sectional, these findings describe associations and do not establish causal direction, and the adjusted estimates are exploratory given the modest sample size. The findings support the routine assessment of caregivers and locally appropriate programs that build practical coping skills and respond to the demands of prolonged care. Larger multicenter longitudinal studies should test causal pathways and determine whether the unexpected positive association between perceived support and burden is reproducible.
